# How to study effectively

**DOI:** 10.1097/IJ9.0000000000000031

**Published:** 2017-06-15

**Authors:** Alexander Fowler, Katharine Whitehurst, Yasser Al Omran, Shivanchan Rajmohan, Yagazie Udeaja, Kiron Koshy, Buket Gundogan

**Affiliations:** a Guy’s and St Thomas’ NHS Foundation Trust; b Royal Devon and Exeter Hospital; c East Anglia NHS Deanery; d Imperial College London; e UCL Medical School, University College London; f Brighton and Sussex University Hospitals; g University College London Medical School, UK

**Keywords:** Study, Medical school, Studying, Examinations

## Abstract

The ability to study effectively is an essential part of completing a medical degree. To cope with the vast amount of information and skills needed to be acquired, it is necessary develop effective study techniques. In this article we outline the various methods students can use to excel in upcoming examinations.

The ability to study effectively is essential in a medical degree. Firstly, having an effective way of learning is key to completing medical school, to cope with the vast volume of information taught. Secondly, medicine and surgery are careers that require constant learning; best practice is ever changing and it is important to be able to integrate these changes into your clinical practice. Thirdly, setting up efficient learning techniques while at medical school will be beneficial to you as and when you approach membership examinations, where study must be fitted around clinical commitments.

Studying effectively depends upon 2 factors: the content you intend to study and how you learn. Learning styles classically fall into 4 groups according to the VARK model (Visual, Aural, Read/Write, and Kinesthetic) but medical students seem to be multimodal in their learning style^1^. This implies that different learning techniques typically ascribed to certain learning styles may be beneficial to students of other learning styles and thus attempting to determine your unique learning style may help to consolidate your methods of study.

Broadly, study is divided into “Book Work” and “Practical Work.” As a medical student, this translates to “Written Papers” and “Clinical Exams,” respectively, although there is often significant overlap. Irrespective of what is to be studied, a plan must be considered first. A solid plan and revision timetable are critical to success upon examination. First, find the date of your examination/s, then work back to deduce how long you have to prepare. At this time, you must also consider the format of the examination, either by reading supplementary material offered by the medical school or by asking for first-hand accounts from students in the years above you who have experienced the examination and can provide extra tips and information. These quick tasks ensure that your preparation and prospective study is well suited to the examination you will do.

Some like to dedicate specific days of the week to certain topics and others, different times in a day and this will vary from person to person. It is possibly best to implement a mixture of the 2, where there is an initial block session to establish the basics, followed by a number of consolidation periods over time to go over and reinforce your learning^2^. For big topics, it is often easier and more time efficient to try and establish a pattern of learning that involves regular, small periods of work. Switching between topics when studying may also aid in effective learning^2^.

## Book work

### Chunking

Chunking is essentially breaking a big topic into smaller components, which are more manageable with regards to study. Depending on the topic, it may be appropriate to break it down in different ways, for example, anatomy may be broken down by location (pelvis/upper limb), whereas pediatrics may be broken down by body system (gastrointestinal/genitourinary). Emergencies can be divided according to the dysfunctional body system or the symptoms that the patient may present with. Chunking allows you to move swiftly between topics. Making links between these different topics or ideas consolidates them within your mind, which also makes information recall easier, a skill much desired for examinations.

### Carrying

It is not unusual to see medical students with small medical pocketbooks on the wards and clinical placements. There are many books that have been made for this intention—to be read when you have a few minutes spare, enough time to read a few key facts but not enough to have a “revision session.” Apps on smart phones also offer a means for this opportunistic learning. It may also be worth carrying some notes with some static points you have to learn, for example, drug doses. Carrying allows you to consolidate your learning around clinical commitments, some of which are often considerably delayed.

### Learning techniques

There is a wide range of techniques people use for their learning:

Studying in different or new locations—Students often have an ideal location where they feel comfortable to study. However, it is proposed that studying in different locations can aid in memory recall and learning^3^,^4^. This could be different areas in the same building or completely separate locations but changing location may reap significant benefits.
Working in groups—Each person may learn a different topic to teach to the student group later. The preparation involved in teaching and the interaction you have with your fellow students can help to consolidate your learning^3^.
Stick men—A simple outline of a stick man can be used and arrows drawn to demonstrate various signs and symptoms of disease. These can aid pattern recognition associated with making diagnoses.
Spider diagrams/mind maps—Mind maps are a revision technique often not utilized by medical students yet they are a good revision technique for enabling factual recall compared with other study methods^5^.
Flash cards—These can be made to cover systems/diseases or specific questions. They are very easy to carry around and can be used alone or as part of a group. There are a number of web programs that create flashcards based on the content you are learning, some of which allow flashcards to be distributed electronically between fellow students^2^,^6^.
Post-it-notes—Around examination times, some students have found sticking post it notes, on which key facts are written, on their walls, desks, or places where they will view them regularly, which may be around the home.

### Resources

It is important that your learning is derived from a range of resources, including past papers. Past papers should be used early to gauge where you are before your revision and then used later when you have covered most of the required material to identify your unique areas of weakness. Online test services and question banks have exploded in recent years. They now enable you to test your knowledge by domain and even by question type (for example, extended matching questions and singe best answers, which each require a different examination technique). Some question banks even offer practice questions outside of the medical degree curriculum but integral for your medical career, such as the examinations, which are done when applying for your foundation post.

It is imperative that the any books and websites you use are up to date with current medical guidelines and best practice. Most medical students use online resources as much, if not more, than book-based resources. Social media, such as Facebook and Instagram, is also rapidly becoming a platform by which you can access medical resources.

## Practical work

The practical examinations of medical school require a slightly different approach to the written examinations. That said, they exist symbiotically, such that a solid basis from your book work will set you up really well for practical examinations. The key to learning for practical examinations (which includes communication history taking, long cases, short cases, clinical examination, and practical procedures) is knowing the format for your examination, and recurrent practice. Recently, practical examinations in medical schools have taken place as OSCEs (Objective Structured Clinical Examination). OSCE practice sessions between students is invaluable and have been shown to provide high-quality feedback when compared with feedback from the senior tutors but it is imperative that the feedback is constructive, recognizing faults with immediate suggestions for improvement^7^,^8^. There are many textbooks available that provide clinical scenarios and mock OSCE stations in order for students to practice among themselves.

### Plan

Planning how to approach your examinations is critical. You should be aware of what you are expected to know, and how you’re going to be assessed on it. The best way to pass practical examinations is to have actually done what they are expecting you to be able to do and receive feedback. For example, if your first time placing a cannula is the week before your examination, you’re unlikely to be as confident as someone doing 5 a week throughout the year during clinical placements. In the lead up to examinations, sketch out a structure of what you need to practice, and how you’re going to do it. It really helps to have a senior colleague who can practice with you—observing and advising as you go.

### Practical skills

This is all practice based so it is best to watch an expert doing it, or access online resources explaining how to do it, then continually practice. Many medical schools will have dedicated teaching sessions run by clinical skill tutors, allowing you learn from a professional approved by the medical school and an opportunity to practice between yourselves, a method found to be very effective^7^. Upon examination, your marks will be based on your technique but also your manner with the supposed patient. You have to be prepared to talk to a mannequin like it is a real patient with the same level of respect.

### Communication skills

There are a number of books detailing how communication skills are examined (the best and most appropriate to use are those written for PACES preparation). The key here is knowing the structures of communication stations, key facts, and practice. Practice with friends going through the scenarios provided. Communication stations take many forms: focused history, explaining a procedure, gaining consent, establishing capacity, explaining a diagnosis, and many others. For history stations, ensure that you have a good structure for your history and key headings, making sure you show empathy for the patient as you go. All communication stations run on a backbone of a clear introduction, good rapport with the patient, and checking for any ideas, concerns, or expectations they may have throughout the consultation.

### Examinations

You must practice examinations with close observation by someone who either examines final year examinations or has recently completed finals/membership examinations. Ideally, set it up so you have short, regular sessions to enable you to develop your technique. Practice needs to be in the time you have allocated, on real patients, with real signs, and with a following presentation/viva as appropriate. Rigorous questioning after each examination regarding patient presentation, diagnosis, investigation, and management can really set you up for any difficult questions that may come up in your end of year OSCE. Around this dedicated practice time, you should be examining every major organ system at least once a week in the build up to finals, and ideally once a month when you are on firms (though this is heavily firm dependent). Some medical schools offer simulation training especially for ABCDE training, a method by which students may feel better prepared for examinations involving problem-solving decisions^9^.

## Looking after yourself

Examination performance, irrespective of the preparation you may have done, is optimized by staying healthy. For many students, examination time translates to prolonged periods of erratic sleep patterns, reduced exercise, poor diet options, and copious amounts of caffeine.

Sleep is key for examination preparation as sleep deprivation reduces the effectiveness of study and can considerably hinder your performance on the day^10^. It is recommended that regular sleep patterns are adopted, aiming for 6 hours of sleep or more daily. Naps in-between study sessions may also aid in effective study. Alongside sleep deprivation, reduced recreational exercise has also been shown to hinder examination performance^11^. It is essential that you keep a schedule of exercise during examination season. Exercising reduces stress, prevents burnout, and delays the onset of mental health conditions such as depression^10^.

A balanced diet must be maintained during examination season. A “bad” diet can affect your energy levels and thus can be detriment to the amount of time you are capable of efficient study. A bad diet may constitute as high energy, high fat with a reduction in protein consumption and this has been shown to correlate to reduced academic performance^12^. Eating a nonbalanced diet may also actually reduce the number of hours of effective study^13^. A balanced diet will also maintain your immune system, to stop you from acquiring an illness, which may slow or effectively halt study and revision times. You must remain hydrated. Being dehydrated is linked to reduced concentration, tiredness, and headaches, none of which constitute to an effective study session.

Finally if you are feeling stressed, you must talk to someone about it. It is most likely that there are numerous other students feeling the same way. You need to ensure you have a robust support network during medical school, particularly around examination time. This network can be made up of family members, fellow students, or friends or the welfare office at your medical school or university. There is a wide range of services that can be offered to you and some of which you can access independently.

## Summary

- Know your examination dates and the amount of time you have to prepare.
- Use a range of learning techniques and study in chunks to ensure effective study sessions.
- Practise for your practical examinations, either with fellow colleagues or sessions run by your medical school.
- Sleep well, exercise, and maintain a healthy, balanced diet especially during examination season.
- Have a robust support network, which can be relied on during periods of stress.
